# Polycomb Group Proteins RING1A and RING1B Regulate the Vegetative Phase Transition in *Arabidopsis*

**DOI:** 10.3389/fpls.2017.00867

**Published:** 2017-05-24

**Authors:** Jian Li, Zheng Wang, Yugang Hu, Ying Cao, Ligeng Ma

**Affiliations:** College of Life Sciences – Beijing Key Laboratory of Plant Gene Resources and Biotechnology for Carbon Reduction and Environmental Improvement, Capital Normal UniversityBeijing, China

**Keywords:** RING1A/B, vegetative phase transition, developmental timing, *SPL*, H2A monoubiquitination, *Arabidopsis*

## Abstract

Polycomb group (PcG) protein-mediated gene silencing is a major regulatory mechanism in higher eukaryotes that affects gene expression at the transcriptional level. Here, we report that two conserved homologous PcG proteins, RING1A and RING1B (RING1A/B), are required for global H2A monoubiquitination (H2Aub) in *Arabidopsis*. The mutation of RING1A/B increased the expression of members of the *SQUAMOSA PROMOTER BINDING PROTEIN-LIKE* (*SPL*) gene family and caused an early vegetative phase transition. The early vegetative phase transition observed in *ring1a ring1b* double mutant plants was dependent on an *SPL* family gene, and the H2Aub status of the chromatin at *SPL* locus was dependent on RING1A/B. Moreover, mutation in RING1A/B affected the miRNA156a-mediated vegetative phase transition, and RING1A/B and the AGO7-miR390-TAS3 pathway were found to additively regulate this transition in *Arabidopsis*. Together, our results demonstrate that RING1A/B regulates the vegetative phase transition in *Arabidopsis* through the repression of *SPL* family genes.

## Introduction

The events of embryonic development are similar between higher animals and plants; however, dramatic differences in post-embryonic development exist. During their life cycle, higher plants undergo three primary post-embryonic developmental transitions, each with a dramatic morphological change (Bäurle and Dean, 2006; Poethig, 2010): the mature seed-to-seedling transition, the juvenile-to-adult phase transition, and the adult vegetative-to-reproductive phase transition. The transition from seed to seedling is referred to as germination, the transition from a juvenile to an adult plant is called the vegetative phase transition, and the transition from vegetative to reproductive growth is called the floral transition, or flowering. The basis for these developmental transitions at the cellular level is cell differentiation, and the basis for cell differentiation at the molecular level is differential gene expression, which is carefully regulated (Bäurle and Dean, 2006; Poethig, 2010). Thus, developmental timing in higher plants is precisely controlled through the regulation of gene expression.

It has been reported that two pathways mediated the vegetative phase transition in *Arabidopsis*. One is the AGO7-miR390-TAS3 pathway, in which AGO7 and miR390 mediated the generation of TAS3 and TAS3 further mediated the cleavage of *ETT* and *ARF4* mRNA to regulate vegetative phase transition (Hunter et al., 2003, 2006; Peragine et al., 2004; Adenot et al., 2006; Fahlgren et al., 2006). While, miR156-mediated cleavage of mRNAs of *SQUAMOSA PROMOTER BINDING PROTEIN-LIKE (SPL)* family genes is another pathway involved in regulating the vegetative phase transition in *Arabidopsis* (Wu and Poethig, 2006; Wu et al., 2009).

Polycomb group (PcG) protein-mediated gene silencing is a major mechanism that regulates gene expression at the transcriptional level in higher eukaryotes (Sawarkar and Paro, 2010; Simon and Kingston, 2013). PcG proteins were initially identified in *Drosophila* through their roles in silencing homeotic genes (Jurgens, 1985). PcG proteins form chromatin-modifying complexes and an epigenetic memory system that mediate transcriptional gene silencing from humans to plants (Sawarkar and Paro, 2010; Mozgova and Hennig, 2015). In mammals, hundreds of genes are silenced by PcG proteins, including genes involved in genomic imprinting, X-inactivation, cell fate transitions, tumorigenesis, and stem cell differentiation (Sauvageau and Sauvageau, 2010; Surface et al., 2010). Studies conducted mostly in *Arabidopsis* have shown that not only is there molecular identity between orthologous PcG proteins from plants and animals, PcG proteins also perform similar developmental and molecular functions, including genomic imprinting, cell fate determination and transitions, and stem cell differentiation (Mozgova and Hennig, 2015; Forderer et al., 2016). Thus, from humans to plants, PcG proteins constitute a global silencing system with key roles in cell differentiation and developmental transitions.

Polycomb group proteins form many different repression complexes (PRCs); however, PRC1 and PRC2 are the main PRCs in animals and plants. In *Arabidopsis*, AtRING1A and AtRING1B (hereafter, RING1A/B) and BMIA/B/C are the central subunits of PRC1, while PRC2 is composed of three complexes, EMF-PRC2, VRN-PRC2, and FIS-PRC2, based on the homologs of animal Su(z)12 in *Arabidopsis* (Bemer and Grossniklaus, 2012). PRC1 (RING1A/B-BMIA/B/C-VALs-ALs) are required for the transition from seed to seedling during germination through the repression of seed maturation genes (Chen et al., 2010; Tang et al., 2012; Yang et al., 2013; Molitor et al., 2014). In comparison, flowering requires the PRC2 complex, which silences the expression of a floral activator, *FT* (Adrian et al., 2010; Wang et al., 2014), or repress the expression of a floral repressor, *FLC* (Sung et al., 2006; Swiezewski et al., 2009; Angel et al., 2011; Coustham et al., 2012; Qüesta et al., 2016). In addition, RING1A is involved in flowering through the repression of *FLC* family genes in *Arabidopsis* (Shen et al., 2014). However, little is known about the regulatory function of PcG proteins in the vegetative phase transition.

Here, we provide genetic, molecular, and biochemical evidence showing that two homologous PcG proteins, RING1A/B, are required for regulation of the vegetative phase transition in *Arabidopsis*. Our data indicate that RING1A/B were found to be required for H2A monoubiquitination (H2Aub) at the chromatin of *SPL* family genes, for the repression of *SPL* gene expression, and for the regulation of miR156 pathway activity. Therefore, RING1A/B repress the vegetative phase transition in plants by regulating *SPL* expression and affect miR156-mediated vegetative phase transition. Thus, this study provides novel insight into the regulation of developmental timing in plants.

## Materials and Methods

### Plant Materials and Growth Conditions

All *Arabidopsis thaliana* genotypes used in this study were in the Columbia background. The *ring1a-2* (SAIL_393_E05), *ring1b-1* (salk_117958), *ring1b-3* (CS874524, SAIL_520_B04), and *zip-1* (CS24281), *spl9-4* (SAIL_150_B05), and *spl15-1* (SALK_074426) mutants were obtained from the ABRC. The *rdr6* and *sgs3* mutants were kindly provided by Dr. Scott Poethig, while *ett* and *afr4* were kindly provided by Dr. Sujuan Cui.

Seeds of all genotypes were sterilized in 1.5% bleach for 10 min, washed with sterilized water three times, kept at 4°C for 2 days, and then germinated on Murashige and Skoog medium containing 1% sucrose. After 10 days of growth in a chamber (160 μmol m^-2^ s^-1^; Percival Scientific Inc., Perry, IA, United States), the plants were transplanted to soil and grown in a growth chamber under LD (16 h of light, 22°C/8 h of dark, 18°C), SD (8 h of light, 22°C/16 h of dark, 18°C), or continuous light conditions at 50% relative humidity.

For the mutant complementation assay, *RING1A* (2,243 bp upstream of the ATG + the gene body + 374 bp downstream of the TGA) or *RING1B* (2,020 bp upstream of the ATG + the gene body + 632 bp downstream of the TAG) genomic DNA was transformed into *ring1a-2 ring1b-3* plants by the floral dip method using *Agrobacterium tumefaciens* (Clough and Bent, 1998). The primers used for constructions are provided in Supplemental Table S1.

### RT-PCR and qRT-PCR

For RT-PCR and qRT-PCR, total RNA was isolated using Trizol reagent (Takara Bio Inc., Otsu, Japan) and then treated with RNase-free DNase (Promega Corp., Madison, WI, United States) to degrade any remaining DNA. Three micrograms of total RNA were used for cDNA synthesis with an oligo(dT) primer and RevertAid^TM^ M-MuLV Reverse Transcriptase (Fermentas, Waltham, MA, United States). RT-PCR was performed with rTaq (Takara Bio Inc.) for limited cycles. qRT-PCR was performed using SYBR Premix Ex Taq (Takara Bio Inc.) in a 7500 Fast Real-Time PCR Instrument (Applied Biosystems, Foster City, CA, United States). *ACTIN7* was included as an internal control. Three biological replicates were performed, with three technical replicates for each. The mean from three biological replicates are shown. The primers used for RT-PCR and qRT-PCR are provided in Supplemental Tables S2, S3.

### Histone Extraction and Western Blotting

Seedlings (2 g, 10 days old, grown on MS plates) were harvested, ground to a powder in liquid nitrogen, and then homogenized in two volumes of lysis buffer (20 mM Tris-HCl, pH 7.4, 25% glycerol, 20 mM KCl, 2 mM EDTA, 2.5 mM MgCl_2_, 250 mM sucrose, 1 mM PMSF, and 5 mM β-mercaptoethanol) at 4°C. The homogenate was filtered through one layer of Miracloth (EMD Millipore, Billerica, MA, United States) and centrifuged at 1,500 × *g* for 10 min at 4°C to pellet the nuclei. The pellet was washed two to four times in nuclei resuspension buffer (20 mM Tris-HCl, pH 7.4, 25% glycerol, and 2.5 mM MgCl_2_) with 0.2% Triton X-100 until the pellet became white or gray, then the pellet was resuspended gently in nuclei resuspension buffer without Triton X-100 and centrifuged at 1,500 × *g* for 10 min at 4°C to pellet the nuclei. The nuclei pellet was treated for 3 h with 0.4 N H_2_SO_4_ and the proteins were precipitated with 25% trichloroacetic acid for 1–2 h. The precipitated proteins were then washed two times with acetone, air-dried, and resuspended in 4 M urea. Equal amounts of histone were subjected to SDS-PAGE, transferred to a PVDF membrane, and probed with anti-ubiquitin (sc-8017; Santa Cruz Biotechnology, Santa Cruz, CA, United States), -H2Aub (8240; Cell Signaling Technology, Danvers, MA, United States), or -H3 antibodies (06-755; EMD Millipore), respectively.

### ChIP

ChIP was performed as described previously (Bowler et al., 2004; Gendrel et al., 2005) using 2 g of 7-day-old seedlings and 6 μl anti-H2Aub antibodies (8240; Cell Signaling Technology). Real-time PCR was performed to quantify the precipitated DNA and input chromatin DNA using SYBR Premix Ex Taq (Takara Bio Inc.) in a 7500 Fast Real-Time PCR Instrument (Applied Biosystems). The primers used for real-time PCR are provided in Supplemental Table S4.

### Small RNA Northern Blotting

Total RNA was isolated using Trizol (Takara Bio Inc.) from various tissues. High molecular weight RNAs were precipitated by incubation in 5% PEG 8000 and 0.5 M NaCl at 4°C for 0.5 h, after which the supernatant was mixed with ∼2.5 volumes of ethanol to precipitate small RNAs. Enriched small RNAs or AGO1-bound RNAs (20 μg) were separated on 7 M urea/15% denaturing polyacrylamide gels and electrically transferred to Hybond-N^+^ nylon membranes (GE Healthcare Life Sciences, Little Chalfont, United Kingdom). The blots were hybridized with LNA probes complementary to miRNAs end-labeled with ^32^P-γ-ATP using T4 polynucleotide kinase (New England Biolabs, Ipswich, MA, United States) at 42°C overnight and washed three times in 1× SSC and 0.1% SDS. The blots were imaged using a Typhoon phosphorimager system (Amersham Biosciences, Little Chalfont, United Kingdom) (Qi et al., 2005). 5S and/or U6 were used as internal loading controls. The probes used for small RNA Northern blotting are provided in Supplemental Table S5.

### Immunopurification of AGO Complexes and Associated Small RNAs

The immunopurification of AGO1 complexes was performed as described previously (Qi et al., 2005). Seedlings (2–4 g, 7 days old, grown on MS plates) grown under SD conditions were collected and ground to a fine powder in liquid nitrogen, and then homogenized in 1 ml/g of extraction buffer (20 mM Tris-HCl, pH 7.5, 100 mM NaCl, 4 mM MgCl_2_, and 0.5% NP-40) containing 5 mM DTT and Complete Protease Inhibitor Cocktail (Roche Life Science, Penzberg, Germany). Cell debris was removed by centrifugation at 14,000 rpm at 4°C for 20 min. The supernatant was collected and pre-cleared by incubation with 20–40 μl of protein A-Sepharose beads (GE Healthcare Life Sciences) at 4°C for 1 h. The pre-cleared extracts were then incubated with anti-AGO1 antibodies (1:50) at 4°C for 2 h. The anti-peptide antibodies for AGO1 were described previously (Mi et al., 2008). The immune complex was collected by incubation with 20–40 μl of protein A-Sepharose beads at 4°C for 1 h and washed three times with extraction buffer. Next, the RNA was extracted from the purified AGO1 complex, and then high molecular weight RNAs were precipitated by incubation in 5% PEG 8000 and 0.5 M NaCl at 4°C for 0.5 h, after which the supernatant was mixed with 2.5 volumes of ethanol to precipitate small RNAs. At last, the small RNAs were resolved by 15% PAGE for Northern blot analysis. 5S and/or U6 were used as internal loading controls.

## Results

### The Mutation of *RING1A/B* Produces an Early Vegetative Phase Transition

RING1A/B are PcG proteins and homologs of human RING1A and RING1B (Xu and Shen, 2008). To examine the function of RING1A/B in *Arabidopsis*, we characterized T-DNA insertion mutants for both genes. One allele for *RING1A* and two alleles for *RING1B* were obtained from the *Arabidopsis* Biological Resource Center (ABRC) (Sessions et al., 2002; Alonso et al., 2003). The *ring1a-2* allele contains a T-DNA insertion within the promoter, 265 bp upstream of the transcription start site, while the *ring1b-1* and *ring1b-3* alleles contain a T-DNA insertion within the second exon and first intron, respectively (Supplemental Figure S1A). These T-DNA insertion sites in *RING1A* and *RING1B* were confirmed in *ring1a* and *ring1b* by sequencing. Full-length transcript accumulation was dramatically decreased in *ring1a-2*, whereas no full-length transcript was detected in *ring1b-1* or *ring1b-3* (Supplemental Figures S1B,C), suggesting that *ring1a-2* is a weak allele, while *ring1b-1* and *ring1b-3* are knockout mutants.

**FIGURE 1:**
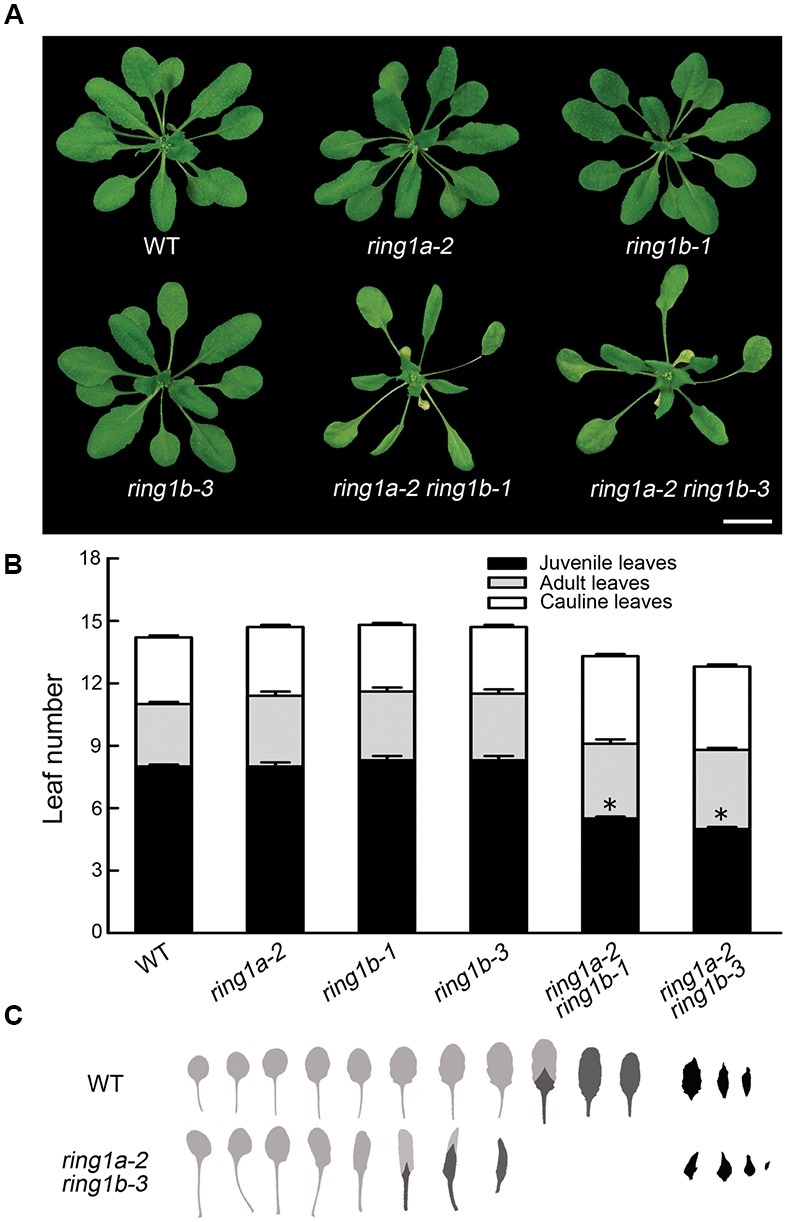
**The mutation of *RING1A/B* causes an accelerated vegetative phase transition in *Arabidopsis*. (A)** Phenotypes of the *ring1a* and *ring1b* plants and their double mutants. Scale bar = 1 cm. **(B)** Vegetative phase transition in wild-type and *ring1a ring1b* double mutant plants. Rosette leaves from 4-week-old wild-type plants, and single and double mutants of *RING1A*/*B* grown under LD conditions are shown. The results represent the mean ± SEM ^∗^ indicates *p* < 0.05. **(C)** Rosette leaf and bract morphology of wild-type and *ring1a-2 ring1b-3* double mutant plants. The leaves are shown in order of their production, from left to right, and shaded to indicate juvenile leaves (light gray), adult leaves (dark gray), or bracts (black).

**FIGURE 2:**
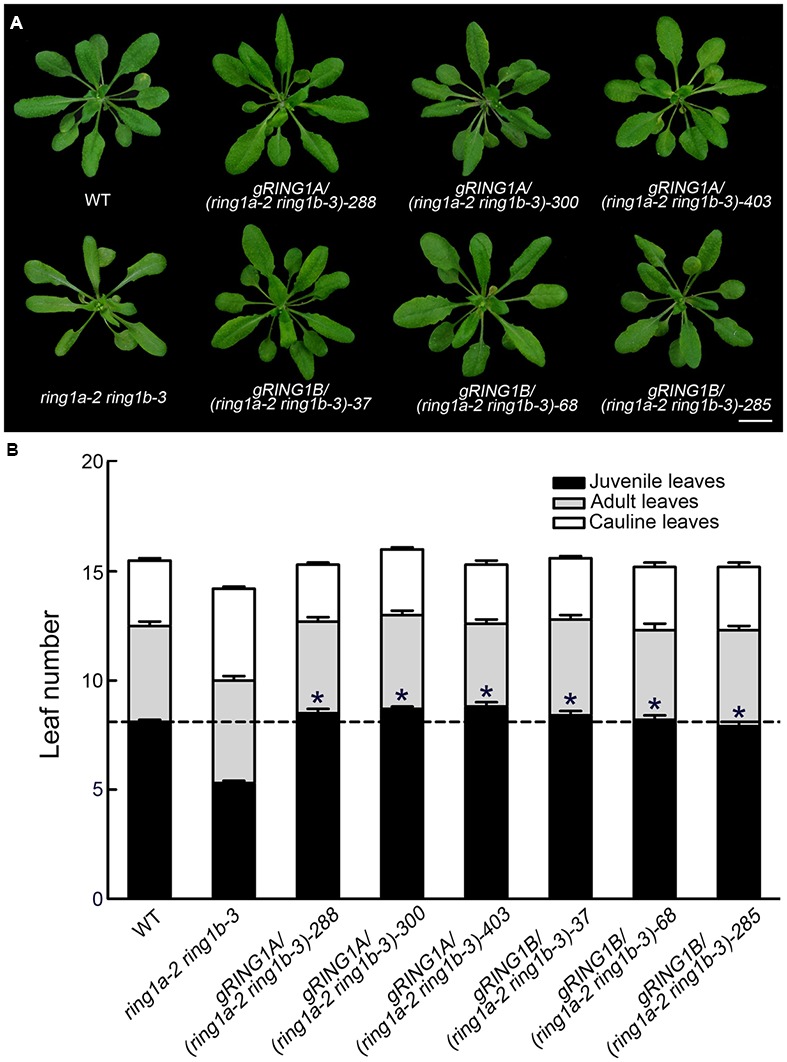
**Complementation of *ring1a-2 ring1b-3* by the transformation of *RING1A* or *RING1B* genomic DNA. (A,B)** The transformation of genomic *RING1A* or *RING1B* DNA into *ring1a-2 ring1b-3* completely complemented the leaf shape **(A)** and early phase transition **(B)** phenotypes observed in *ring1a-2 ring1b-3* plants. The result represents the mean ± SEM, *n* > 15. Plants of WT, *ring1a-2 ring1b-3*, and the complemented lines were grown under LD conditions for 4 weeks. Of 25 lines examined, 24 were rescued by the transformation of *RING1A*, while all 24 lines examined were rescued by the transformation of *RING1B*. T2 generation of transgenic plants was used. Three representative lines of each type are shown. Scale bar = 1 cm. The results represent the mean ± SEM. ^∗^*p* < 0.05.

Neither the *RING1A* nor the *RING1B* single mutant exhibited an obvious phenotype, while the double mutants, *ring1a-2 ring1b-1* and *ring1a-2 ring1b-3*, in addition to the late flowering phenotype in term of the days to flowering (**Table 1**), displayed similar phenotypes with downward curling leaves (**Figure 1A**), which in the mutant plants is present in early development (Telfer et al., 1997), suggesting a vegetative phase transition defect in *ring1a ring1b*. To verify this, we examined the appearance of abaxial trichomes during leaf development, which is a key trait of adult leaves (Telfer et al., 1997). The double mutant (*ring1a ring1b*) showed reduced numbers of leaves without abaxial trichomes (about 2, 2.5, and 5) compared to wild-type plants under constant light, long-day (LD), and short-day (SD) conditions, respectively (**Figures 1B,C** and **Table 1**). The transformation of genomic *RING1A* or *RING1B* into *ring1a ring1b* plants completely complemented the leaf shape and abaxial trichome appearance phenotypes observed in the double mutant (**Figures 2A,B**). These results indicate that the mutation of *RING1A/B* caused the precocious appearance of adult leaf traits, and that RING1A/B are functionally redundant in the regulation of the vegetative phase transition in *Arabidopsis.*

**Table 1:** The mutation of both *RING1A* and *RING1B* produced an early vegetative phase change under different photoperiods.

Photoperiod	Genotype	Number of leaves without trichomes	Number of leaves with trichomes	Number of rosette leaves	Number of cauline leaves	Days to a visible bud	*n*
Continuous light	WT	7.5 ± 0.2	2.9 ± 0.2	10.3 ± 0.2	2.8 ± 0.1	22.0 ± 0.3	29
	*ring1a-2*	7.3 ± 0.2	3.4 ± 0.2	10.7 ± 0.2	2.6 ± 0.2	23.7 ± 0.2	22
	*ring1b-1*	7.3 ± 0.2	3.0 ± 0.2	10.2 ± 0.2	2.5 ± 0.1	23.1 ± 0.2	20
	*ring1b-3*	7.4 ± 0.2	3.3 ± 0.2	10.7 ± 0.3	2.6 ± 0.2	23.3 ± 0.3	18
	*ring1a-2 ring1b-1*	5.6 ± 0.1^a^	3.4 ± 0.2	9.0 ± 0.2	4.0 ± 0.2	25.1 ± 0.3	20
	*ring1a-2 ring1b-3*	5.5 ± 0.1^a^	3.2 ± 0.2	8.7 ± 0.1	3.9 ± 0.1	25.8 ± 0.2	21
Long days	WT	8.0 ± 0.1	3.0 ± 0.1	11.0 ± 0.1	3.2 ± 0.1	24.1 ± 0.2	20
	*ring1a-2*	8.0 ± 0.2	3.4 ± 0.2	11.4 ± 0.2	3.3 ± 0.1	24.6 ± 0.4	20
	*ring1b-1*	8.3 ± 0.2	3.3 ± 0.2	11.6 ± 0.3	3.2 ± 0.1	24.7 ± 0.6	18
	*ring1b-3*	8.3 ± 0.2	3.2 ± 0.2	11.5 ± 0.2	3.2 ± 0.1	24.8 ± 0.5	18
	*ring1a-2 ring1b-1*	5.5 ± 0.1^a^	3.6 ± 0.2	9.0 ± 0.2	4.2 ± 0.1	25.7 ± 0.3	24
	*ring1a-2 ring1b-3*	5.0 ± 0.1^a^	3.8 ± 0.1	8.8 ± 0.2	4.0 ± 0.1	27.0 ± 0.2	24
Short days	WT	16.9 ± 0.2	31.8 ± 0.6	48.7 ± 0.7	8.1 ± 0.2	75.0 ± 1.1	24
	*ring1a-2 ring1b-1*	10.5 ± 0.2^a^	26.7 ± 0.7	37.2 ± 0.7	11.0 ± 0.2	84.3 ± 1.1	21
	*ring1a-2 ring1b-3*	9.2 ± 0.2^a^	23.1 ± 0.8	32.3 ± 0.8	13.3 ± 0.3	98.3 ± 1.2	24

*^a^Significantly different from wild-type plants (WT; p < 0.05 t-test). All values are means ± SEM.*

### RING1 and the AGO7-miR390-TAS3 Pathway Additively Regulate the Vegetative Phase Transition in *Arabidopsis*

**FIGURE 3:**
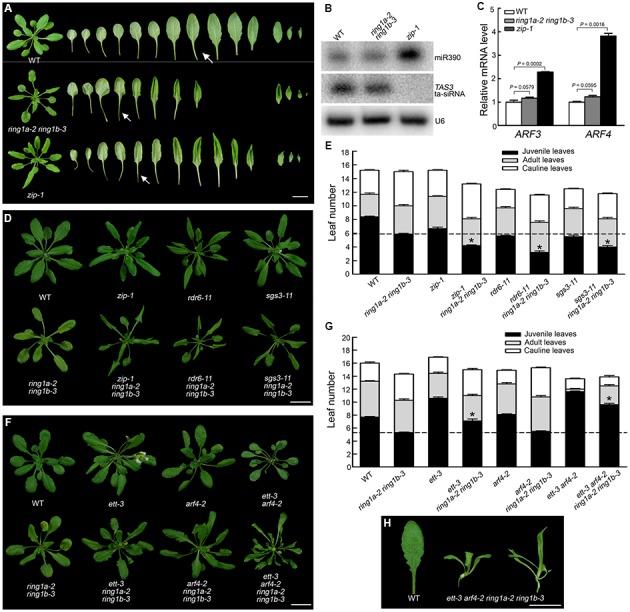
**RING1A/B and the AGO1-miR390-TAS3 pathway additively regulate the vegetative phase transition in *Arabidopsis*. (A)** Phenotypes of the *ring1a ring1b* and *zip-1* plants. Wild-type, *ring1a-2 ring1b-3*, and *zip-1* plants were grown under LD conditions for 4 weeks. Scale bar = 1 cm. **(B)** Northern blot analysis of small RNAs from the shoot apex of SD-grown 16-day-old wild-type, *ring1a-2 ring1b-3*, and *zip-1* plants hybridized with probes against miR390 and *TAS3* tasi-RNA. U6 was used as a loading control. **(C)** Analysis of the relative expression levels of *ARF3* and *ARF4* in the shoot apex of 16-day-old wild-type, *ring1a-2 ring1b-3*, and *zip-1* plants grown under SD conditions by qRT-PCR. The levels were normalized to wild-type plants using *ACTIN7* as an internal control. The result represents the mean + SEM of three independent experiments. *p*-values of relative mRNA level in *ring1a-2 ring1b-3* or *zip-1* versus wild-type were indicated. **(D,E)** Phenotypes of the *ring1a ring1b*, *zip-1*, *rdr6*, and *sgs3* plants and their triple mutants. Plants of WT, *ring1a-2 ring1b-3*, *zip-1*, *rdr6-11*, and *sgs3-11* and their triple mutants were grown under LD conditions for 4 weeks. Scale bar = 1 cm in **(D)**. The result represents the mean ± SEM in **(E)**. ^∗^*p* < 0.05. **(F,G)** Phenotypes of *ring1a ring1b*, *ett3*, and *arf4* and of their triple and quadruple mutant plants. Plants of WT, *ring1a-2 ring1b-3*, *ett3*, and *arf4* and their triple and quadruple mutant plants were grown under LD conditions for 4 weeks. Scale bar = 1 cm in **(F)**. The results represent the mean ± SEM in **(G)**. ^∗^*p* < 0.05. **(H)** Leaf phenotype observed in *ring1a-2 ring1b-3 ett3 arf4*. Scale bar = 1 cm.

The phenotype of the *ring1a ring1b* plants was similar to that of *zip* (*ago7*) (**Figure 3A**), which exhibits an early vegetative phase transition and defects in the tasiRNA-TAS3 pathway (Hunter et al., 2003, 2006; Peragine et al., 2004; Adenot et al., 2006; Fahlgren et al., 2006). To determine whether RING1A/B are involved in the AGO7-miR390-TAS3 pathway, we first examined the levels of *TAS3* tasiRNA and miR390 by Northern blotting and the levels of their target genes *AFR3/ETT* and *ARF4* by quantitative (q)RT-PCR in *ring1a ring1b* and wild-type plants, using *zip-1* as a positive control. There was no obvious change in the expression levels of miR390, *TAS3*, *ARF3/ETT*, and *ARF4* in *ring1a ring1b*; in contrast, the expression of miR390, *ARF3/ETT*, and *ARF4* was up-regulated while that of *TAS3* was down-regulated in *zip-1* (**Figures 3B,C**), suggesting that RING1A/B are not involved in the AGO7-miR390-TAS3 pathway.

We also observed that mutations in components of the AGO7-miR390-TAS3 pathway, including ZIP, RDR6, and SGS3, produced an early vegetative phase transition phenotype (**Figures 3D,E**), consistent with previous reports (Hunter et al., 2003, 2006; Peragine et al., 2004; Adenot et al., 2006; Fahlgren et al., 2006). However, triple mutant plants (*ring1a ring1b zip*, *ring1a ring1b rdr6*, and *ring1a ring1b sgs3*) exhibited a stronger early vegetative phase transition phenotype than *ring1a ring1b*, *zip*, *rdr6*, or *sgs3* mutant plants (**Figures 3D,E**). Furthermore, the mutation of ARF3/ETT and ARF4 suppressed the early vegetative phase transition phenotype observed in *ring1a ring1b*, as the vegetative phase transition in *ring1a ring1b ett arf4* was later than that in wild-type plants and in between that in *ring1a ring1b* and *ett arf4* double mutant plants (**Figures 3F,G**). Moreover, the *ring1a ring1b ett arf4* plants had compound leaves while the *ring1a ring1b* and *ett arf4* double mutant and *ring1a ring1b ett* and *ring1a ring1b arf4* triple mutant plants had simple leaves (**Figures 3F,H**). These results suggest that RING1A/B and the AGO7-miR390-TAS3 pathway additively regulate the vegetative phase transition in *Arabidopsis*.

### The Mutation of *RING1A*/*B* Causes Increased *SPL* Gene Expression

**FIGURE 4:**
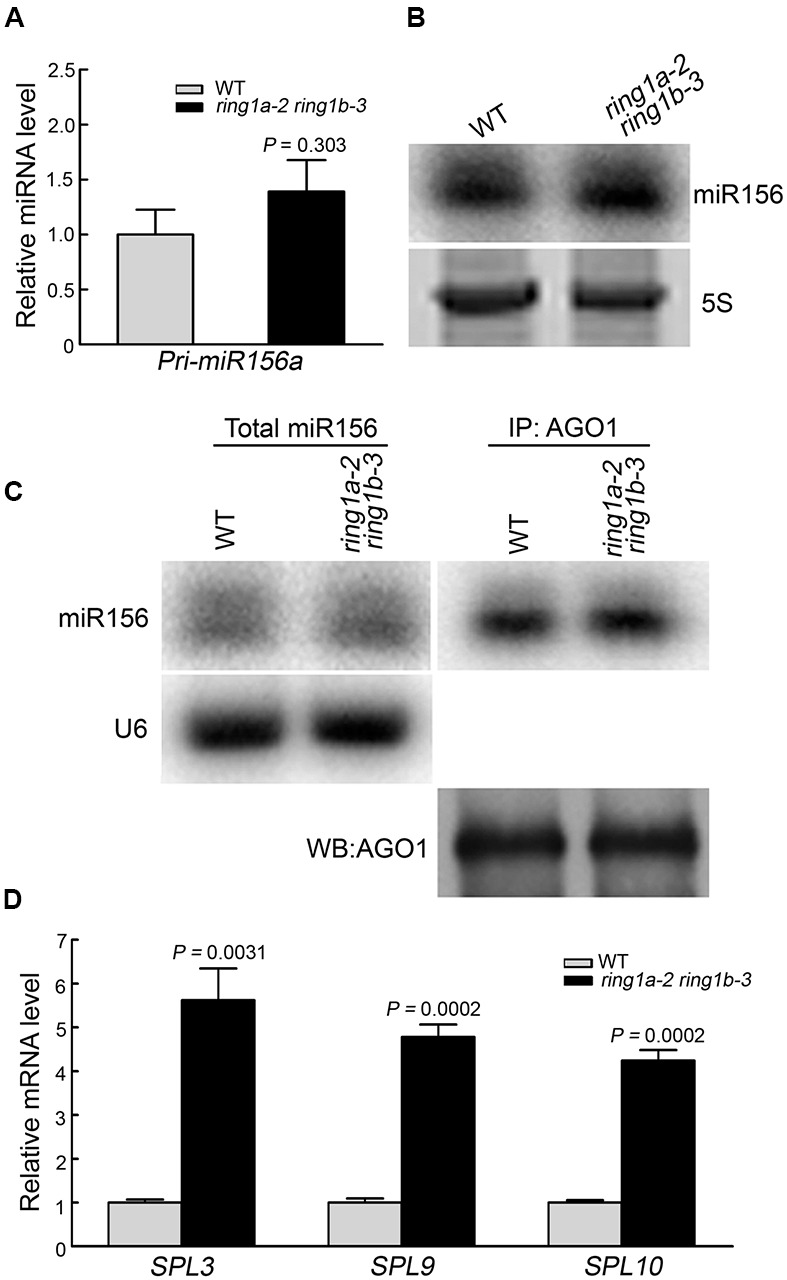
**The mutation of RING1A/B increased *SPL* family gene expression. (A)** qRT-PCR analysis of the pri-miR156a a level in 5-day-old wild-type and *ring1a-2 ring1b-3* seedlings grown under SD conditions. The levels were normalized to that in WT using *ACTIN7* as an internal control. The result represents the mean ± SEM of three independent experiments. *p*-values of relative miRNA level in *ring1a-2 ring1b-3* versus wild-type were indicated. **(B)** Northern blotting of the total miR156 levels in wild-type and *ring1a-2 ring1b-3* seedlings grown under SD conditions for 7 days. 5S RNA was used as a loading control. Three independent biological replicates were conducted, and one representative result was shown. **(C)** Northern blotting of the miR156 level in total small RNA (total miR156) and immunoprecipitated small RNA preparations using anti-AGO1 antibodies (AGO1-bound miR156). U6 was used as a loading control for total small RNA. Precipitated AGO1 is shown in the bottom panel. WB, Western blotting. Four independent biological replicates were conducted, and one representative result was shown. **(D)** qRT-PCR analysis of three *SPL* family genes in 7-day-old wild-type and *ring1a-2 ring1b-3* plants grown under SD conditions. The levels in **(D)** were normalized to that in WT; *ACTIN7* was used as an internal control. The results represent the mean ± SEM of five independent experiments. *p*-values of relative mRNA level in *ring1a-2 ring1b-3* versus wild-type were indicated.

It was previously reported that miR156 is involved in regulating the vegetative phase transition in *Arabidopsis* by targeting *SPL* family genes (Wu and Poethig, 2006; Wu et al., 2009). To examine whether RING1A/B are involved in the regulation of miR156 function, we examined the levels of pri-miR156a and mature miR156 in *ring1a ring1b* and wild-type plants by qRT-PCR and Northern blotting, respectively. There was a slight but non-significant (*p* = 0.3) increase in the level of pri-miR156a in *ring1a ring1b* plants, and no obvious difference in the total mature miR156 level between *ring1a ring1b* and wild-type plants (**Figures 4A,B**). The latter was confirmed in an additional eight replicates (data not shown). In addition, there was no obvious difference in the amount of AGO1-bound miR156 between *ring1a ring1b* and wild-type plants (**Figure 4C**). These data suggest that the level of mature miR156 is no obviously regulated by RING1A/B.

We also investigated whether RING1A/B are involved in the regulation of *SPL* expression. For this purpose, we examined the expression of the representative members of the *SPL* family (*SPL3*, *SPL9*, and *SPL10*) involved in vegetative phase transition, and we found that the expression of *SPL3*, *SPL9*, and *SPL10* were up-regulated in *ring1a ring1b* plants compared to wild type (**Figure 4D**). This result indicates that the mutation of *RING1A*/*B* increased the expression of *SPL* family genes.

### RING1A/B Mediate the Vegetative Phase Transition and Leaf Emergence in *Arabidopsis* in an *SPL*-Dependent Manner

**FIGURE 5:**
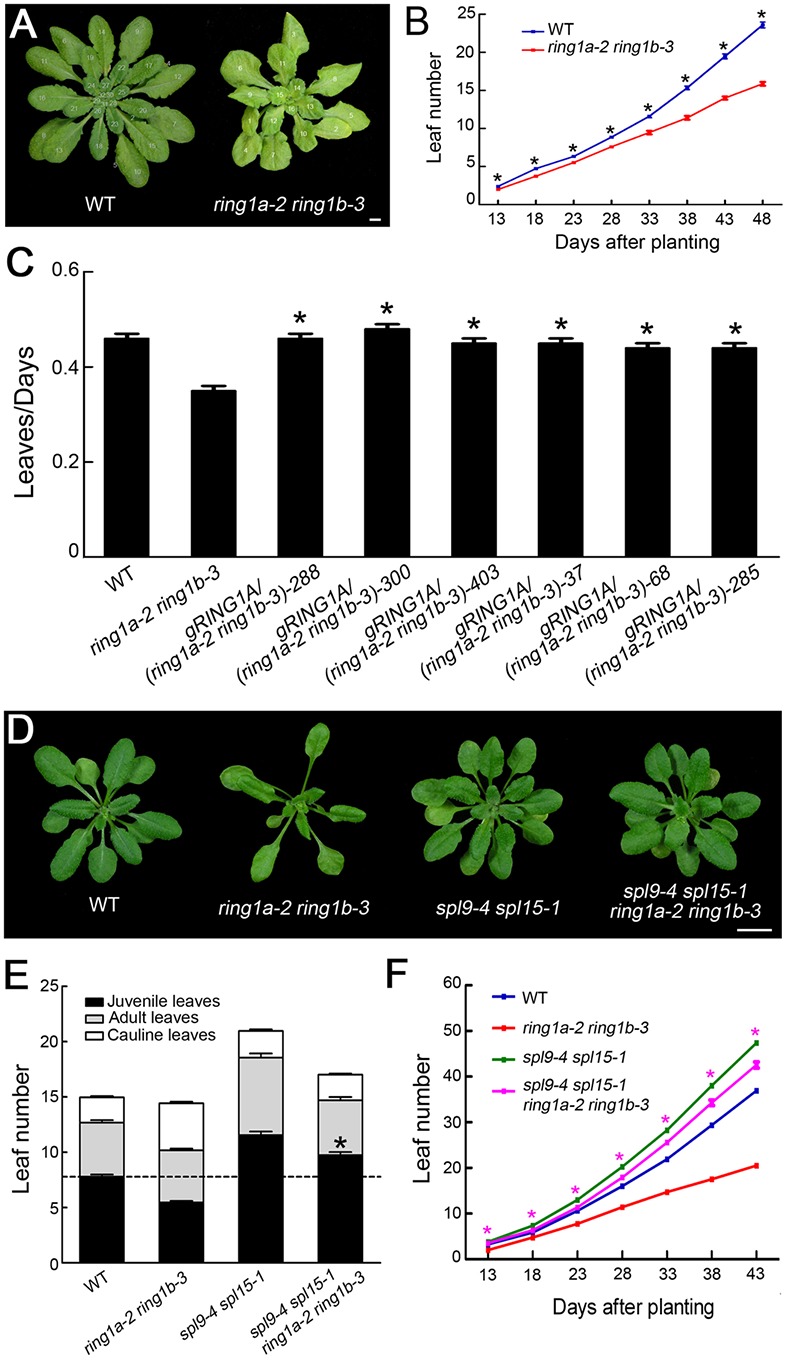
**The early vegetative phase transition and decreased leaf emergence phenotypes in *ring1a-2 ring1b-3* are dependent on *SPL* family genes. (A,B)** The rate of leaf emergence in wild-type and *ring1a-2 ring1b-3* plants grown under SD conditions. The results represent the mean ± SEM, *n* = 15. ^∗^*p* < 0.05. **(C)** The transformation of genomic *RING1A* or *RING1B* DNA into *ring1a-2 ring1b-3* completely complemented the leaf emergence phenotype of the mutant. Plants of WT, *ring1a-2 ring1b-3*, and the complemented lines were grown under SD conditions for 4 weeks. Of 25 lines examined, 24 were rescued by the transformation of *RING1A*, while all 24 lines examined were rescued by the transformation of *RING1B*. The result represents the mean ± SEM, *n* > 15. ^∗^*p* < 0.05. **(D)** The phenotypes of *ring1-2a ring1b-3*, *spl9-4 spl15-1*, and their quadruple mutant plants. Scale bar = 1 cm. **(E)** The numbers of juvenile leaves, adult leaves and cauline leaves in wild-type, *ring1-2a ring1b-3*, and *spl9-4 spl15-1* plants and the quadruple mutants. The result represents the mean ± SEM of three independent experiments. The result represents the mean ± SEM. ^∗^*p* < 0.05, *n* > 15. **(F)** The rate of leaf emergence in wild-type, *ring1-2a ring1b-3*, and *spl9-4 spl15-1* plants and in the quadruple mutant plants grown under SD conditions. The result represents the mean ± SEM of three independent experiments. ^∗^*p* < 0.05.

In addition to affecting the vegetative phase transition, the mutation of *RING1A/B* decreased the rate of leaf emergence (**Figures 5A,B**). The decrease in leaf emergence in *ring1a ring1b* was completely complemented by the transformation of *RING1A* or *RING1B* into *ring1a ring1b* plants (**Figure 5C**), indicating that leaf emergence is mediated by RING1A/B in *Arabidopsis*.

*SPL* family genes are involved in regulating both the vegetative phase transition and leaf emergence in *Arabidopsis* (Schwab et al., 2005; Wang et al., 2008; Wu et al., 2009). Thus, to examine whether RING1A/B mediate the vegetative phase transition and leaf emergence by repressing the expression of *SPL* family genes, we generated *spl9 spl15* double mutant and *ring1a ring1b spl9 spl15* quadruple mutant plants. The *spl9 spl15* plants exhibited a delayed vegetative phase transition and increased leaf emergence compared to wild-type plants (**Figures 5D–F**). In addition, the early vegetative phase transition and decrease in leaf emergence in *ring1a ring1b* were suppressed in *ring1a ring1b spl9 spl15* plants (**Figures 5D–F**), suggesting that the effects of RING1A/B on the regulation of the vegetative phase transition and leaf emergence are dependent on *SPL* family genes.

### RING1A/B Control the H2Aub of *SPL* Gene Chromatin

**FIGURE 6:**
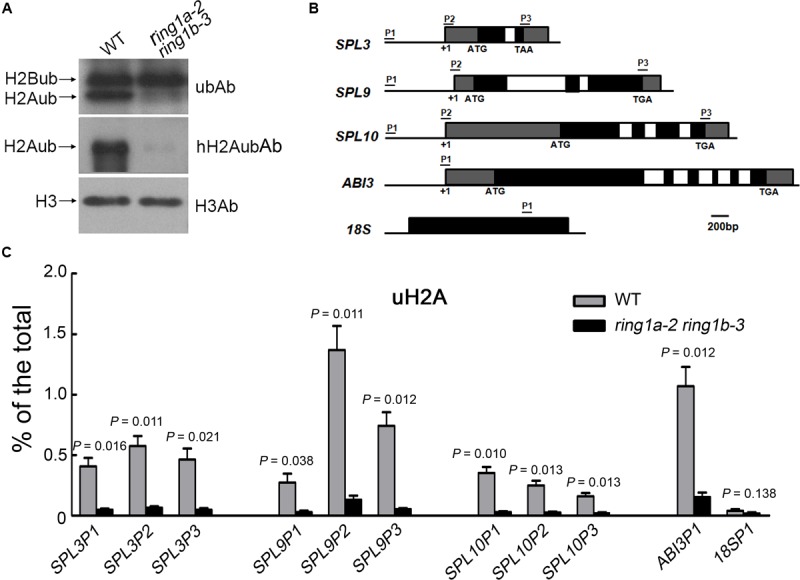
**H2Aub of *SPL* family gene chromatin is dependent on RING1A/B. (A)** The mutation of RING1A/B caused a defect in global H2Aub. H3 was used as a loading control. **(B)** Schematic diagram of the *SPL*s, *ABI3* and *18S* loci indicating the regions analyzed by qRT-PCR after ChIP. The filled black boxes represent exons, the open boxes represent introns, and filled gray boxes represent untranslated regions. +1, the transcription initiation point. **(C)** H2Aub at *SPL* family gene chromatin in wild-type and *ring1a-2 ring1b-3* plants. The level of H2Aub at *SPL*s chromatin was assessed by qRT-PCR after ChIP using anti-H2Aub antibodies. The data represent the amount of immunoprecipitated chromatin versus the total amount of chromatin used. Error bars indicate the SEM of three independent experiments. The *ABI3* gene was used as a positive control, and 18S rRNA gene was used as a negative control. *p*-values of % of the total in *ring1a-2 ring1b-3* versus wild-type were indicated.

PRC1 mediates H2Aub in *Arabidopsis* (Bratzel et al., 2010; Li et al., 2011; Yang et al., 2013). To determine whether RING1A/B are required for H2Aub, we first examined the global levels of H2Aub in wild-type and *ring1a ring1b* plants. The mutation of RING1A/B had no effect on H2B monoubiquitination (H2Bub), but it noticeably decreased the global level of H2Aub (**Figure 6A**), indicating that RING1A/B are required for H2Aub in *Arabidopsis*.

To determine whether the H2A in *SPL* chromatin is monoubiquitinated and whether the H2Aub of *SPL* chromatin is mediated by RING1A/B, we measured the level of H2Aub in *SPL* gene chromatin in both wild-type and *ring1a ring1b* plants by ChIP assays. Monoubiquitination of H2A was identified in *SPL* gene chromatin, and the level of H2Aub in the chromatin of *SPL3*, *SPL9*, and *SPL10* was obviously decreased in *ring1a ring1b* compared to that in wild-type plants (**Figures 6B,C**). These results suggest that H2Aub in the chromatin of *SPL3*, *SPL9*, and *SPL10* is mediated by RING1A/B.

### Regulation of the Vegetative Phase Transition by miR156 Is Affected by RING1A/B

**FIGURE 7:**
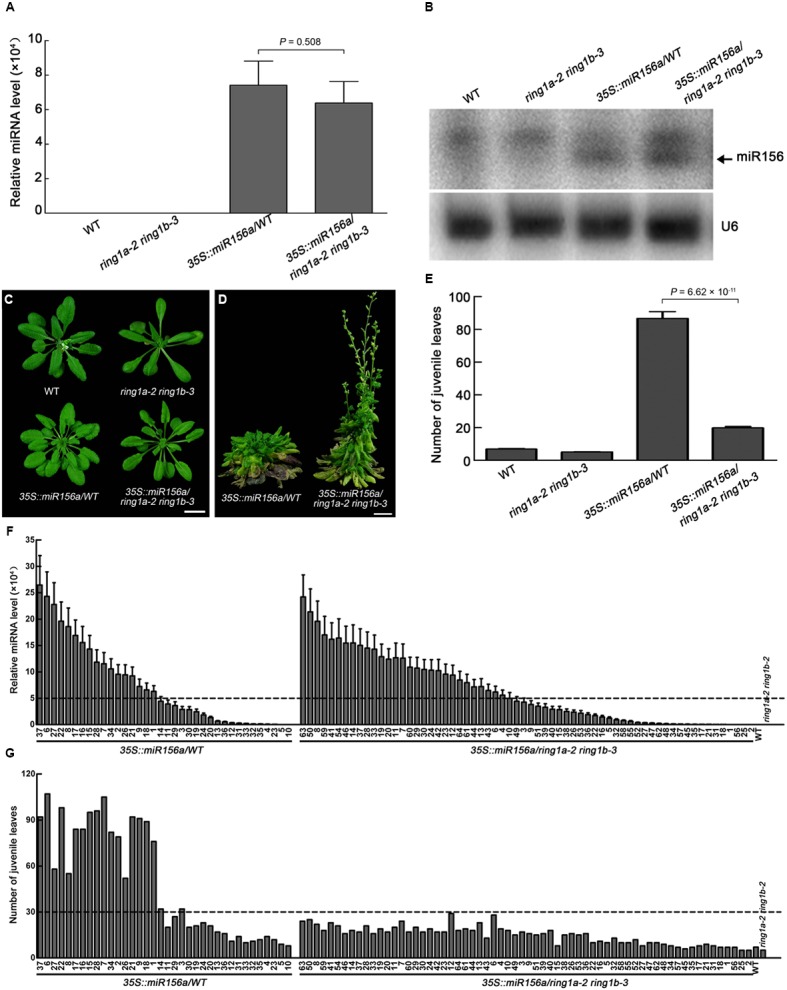
**The efficiency of miR156 in the vegetative phase transition is dependent on RING1A/B. (A)** Measurement of the pri-miR156a level in wild-type, *ring1a-2 ring1b-3*, *35S::miR156a* in wild-type (*35S::miR156/*WT), and *35S::miR156a* in *ring1a-2 ring1b-3* (*35S::miR156a*/*ring1a-2 ring1b-3*) plants by qRT-PCR. The leaves of 4-week-old plants grown under LD conditions were used. The levels were normalized to that in WT using *ACTIN7* as an internal control. The result represents the mean ± SEM from 36 individual plants for *35S::miR156a/*WT and 64 individual plants for *35S::miR156a/ring1a ring1b*. **(B)** Detection of the level of miR156a in wild-type, *ring1a-2 ring1b-3*, *35S::miR156a*/WT, and *35S::miR156a*/*ring1a-2 ring1b-3* plants by Northern blotting. Leaves from 4-week-old plants grown under SD conditions were used. U6 RNA was used as a loading control. Two independent biological replicates were conducted, and one representative result was shown. **(C,D)** Phenotypes of 4-week-old **(C)** and 12-week-old **(D)** *ring1a-2 ring1b-3*, *35S::miR156a*/WT, and *35S::miR156a*/*ring1a-2 ring1b-3* plants grown under LD conditions. Scale bar = 1 cm. **(E)** Vegetative phase transition phenotypes in *ring1a-2 ring1b-3*, *35S::miR156a*/WT, and *35S::miR156a*/*ring1a-2 ring1b-3* plants. The result represents the mean ± SEM from*35S::miR156a/*WT (*n* = 36) and *35S::miR156a/ring1a ring1b* (*n* = 64). The data shown for *35S::miR156a*/WT are from 36 independent transgenic plants, while the data shown for *35S::miR156a*/*ring1a-2 ring1b-3* are from 64 independent transgenic plants. **(F)** The distribution of expression level of pri-miR156a from independent heterozygous transgenic plants for *35S::miR156a*/WT (*n* = 36) and *35S::miR156a*/*ring1a-2 ring1b-3* (*n* = 64). The result represents the mean + SEM of three replicates. The level of pri-miR156a was measured by qRT-PCR; the levels were normalized to that in WT using *ACTIN7* as an internal control. The pri-miR156a levels in wild-type and *ring1a-2 ring1b-3* plants were used as positive controls. The leaves of 4-week-old plants grown under LD conditions were used. **(G)** The distribution of number of juvenile leaves from independent heterozygous transgenic plants for *35S::miR156*/WT (*n* = 36) and *35S::miR156a*/*ring1a-2 ring1b-3* (*n* = 64). The number of juvenile leaves in wild-type and *ring1a-2 ring1b-3* plants were used as positive controls.

MiR156 represses the vegetative phase transition by targeting mRNAs produced from *SPL* family genes for degradation. An increase in *SPL* expression may therefore suppress the miR156-repressed vegetative phase transition. To determine whether the mutation of RING1A/B affects the efficiency of the regulation of the vegetative phase transition by miR156, we overexpressed miR156a in wild-type and *ring1a ring1b* plants under the control of the 35S promoter. MiR156 overexpression in wild-type plants significantly delayed the vegetative phase transition (Wu et al., 2009). To ensure that miR156 was overexpressed, we measured the level of miR156 by both Northern blotting using mixed samples from all representative transgenic plants and qRT-PCR using individual representative transgenic plants (36 *35S::miR156a/*WT plants and 64 *35S::miR156a/ring1a ring1b* plants). MiR156 was overexpressed in almost all representative individual transgenic plants, and there was no obvious difference in the level of miR156 between the *35S::miR156a/*WT and *35S::miR156a/ring1a ring1b* plants (**Figures 7A,B**). However, even though the vegetative phase transition was delayed in *35S::miR156a/ring1a ring1b* compared to wild-type plants, it occurred much earlier than in *35S::miR156a/*WT plants (**Figures 7C–F**). These results indicate that the miR156-regulated vegetative phase transition is affected by RING1A/B.

## Discussion

RING1 is a component of PRC1 in *Arabidopsis*. It is required for the transition from the embryonic stage to the vegetative stage of development, and for the transition from the adult stage to the reproductive stage. In this study, we verified that RING1A/B repress the juvenile-to-adult transition by regulating *SPL* gene expression. In addition, RING1A/B also affect miR156-regulated vegetative phase transition through an unknown way in *Arabidopsis*.

### RING1A/B Repress the Vegetative Phase Transition

The vegetative phase transition is a key change during higher plant development; it links the adult and reproductive stages of the life cycle (Bäurle and Dean, 2006; Poethig, 2010). Upon completing the vegetative phase transition, plants become competent to flower; flowering leads to seed production, and enables plants to complete their life cycle under suitable environmental conditions.

Xu and Shen (2008) used a strong *ring1a* allele to study the functions of RING1A/B, and they found that RING1A/B are required for meristem function; *ring1a ring1b* plants exhibited defects in leaf and stem shape, as well as in floral organ number. They suggested that the release of *KNOX* genes in rosette leaves may account for the defects in *ring1a ring1b* (Xu and Shen, 2008). As the double knockout mutant exhibited drastic growth defects and was completely sterile, we could not explore the function of RING1 in other developmental processes; thus, we used a weak *ring1a* allele and *ring1b* knockout mutants to generate double mutants in order to analyze the functions of RING1A/B at all stages of *Arabidopsis* development.

In an analysis of the key trait (abaxial trichrome distribution), we found that two double mutants produced from plants expressing a weak *ring1a* and two *ring1b* knockout alleles (*ring1a-2 ring1b-1* and *ring1a-2 ring1b-3*) exhibited similar early vegetative phase transition phenotypes (**Figure 1**), and that these phenotypes were fully complemented when *RING1A* or *RING1B* was transformed into *ring1a-2 ring1b-1* or *ring1a-2 ring1b-3* plants (**Figure 2**). Thus, our results suggest that RING1A/B function in the repression of the vegetative phase transition in *Arabidopsis*. We further found that the H2A in *SPL* gene chromatin was monoubiquitinated, and that the level of H2Aub in *SPL* locus was dependent on RING1A/B (**Figure 6C**). In addition, *SPL* gene expression was up-regulated in *ring1a ring1b* plants (**Figure 4D**). Our results indicate that RING1A/B may repress the vegetative phase transition through the modification of H2A in *SPL* gene chromatin and the repression of *SPL*s expression.

### RING1A/B Affects miR156-Mediated Vegetative Phase Transition

The vegetative phase transition is controlled via at least two pathways: the AGO7-miR390-TAS3 pathway and miR156 pathway (Poethig, 2009). The phenotype of the *ring1a ring1b* plants was similar to that of *ago7* (*zip-1*) in terms of leaf shape (downward curling leaves), petiole length (long petioles), and abaxial trichrome distribution (early appearance of trichomes on the underside of leaves) (**Figures 3A,D,E**). However, no change was detected in the levels of miR390, TAS3 tasiRNA, and their targets *ARF3* and *ARF4* in *ring1a ring1b* plants compared to wild-type plants (**Figures 3B,C**). In addition, the early vegetative phase transition phenotype of triple mutants of *ring1a ring1b* and AGO7-miR390-TAS3 pathway components (e.g., *zip*, *rdr6*, and *sgs3*) was more severe than that in *ring1a ring1b* and any of the single AGO7-miR390-TAS3 pathway component mutants (**Figures 3D,E**). Furthermore, the early vegetative phase transition phenotype of *ring1a ring1b arf3 arf4* was weaker than that of *arf3 arf4* but more severe than that of *ring1a ring1b* (**Figures 3F,G**). These results indicate that RING1A/B and AGO7-miR390-TAS3 are not part of the same pathway but that they additively regulate the vegetative phase transition in *Arabidopsis*.

The miR156-mediated microRNA pathway is another key pathway that regulates the vegetative phase transition; miR156 targets mRNAs encoded by *SPL* family genes for degradation to repress the vegetative phase transition (Wu and Poethig, 2006; Wu et al., 2009). Consistent with previous reports, miR156 overexpression caused an obvious delay in the vegetative phase transition (Wu and Poethig, 2006; Wu et al., 2009) (**Figure 7**). However, the ability with which miRNA156 delayed the vegetative phase transition was obviously decreased in *ring1a ring1b* compared to wild-type plants (**Figure 7**). These data suggest that RING1A/B might affect the processing, stability or function of miRNA156 during the vegetative phase transition. Alternatively, we cannot rule out the possibility that RING1A/B act independently of miRNA156.

### PcG Proteins Mediate the Juvenile-to-Adult Phase Change in Plants

Recent studies have suggested that PcG proteins are required for the transition from embryonic to vegetative growth and the transition from adult to reproductive growth (Pu and Sung, 2015; Xiao and Wagner, 2015). However, little is known about whether PcG proteins are involved in regulating the juvenile-to-adult phase transition in plants. Picó et al. (2015) reported that BMIA/B promoted the juvenile-to-adult phase transition via the direct repression of pri-miR156 expression in *Arabidopsis*; the vegetative phase transition was delayed and pri-miR156 expression was up-regulated in *bmi1a bmi1b* plants. Xu M. et al. (2016) reported that SWN, a component of PRC2, worked in parallel to chromatin remodeler PKL to promote vegetative phase transition, and Xu Y. et al. (2016) reported that SWN act antagonistically with a chromatin remodeler BRM to regulate vegetative phase change. In the present study, RING1A/B repressed the juvenile-to-adult phase transition in *Arabidopsis* (**Figures 1**, **2**). The levels of pri-miR156, mature miR156, and AGO1-bound active miR156 were not obviously changed in *ring1a ring1b* compared to wild-type plants (**Figures 4A–C**). However, *SPL* family gene expression was up-regulated in *ring1a ring1b* (**Figures 4D,E**), and the efficiency of miR156 in the regulation of the vegetative phase transition was decreased in *ring1a ring1b* (**Figure 7**).

RING1A/B and BMIA/B are thought to be components of the PRC1 complex in *Arabidopsis* (Sanchez-Pulido et al., 2008; Xu and Shen, 2008; Bratzel et al., 2010). However, these PRC1 components mediate the vegetative phase transition in opposing ways by targeting different genes. Xu M. et al. (2016) also reported that mutation in SWN leads to the delayed vegetative phase change, while mutation in CLF, a homolog of SWN and component of PRC2, leads to early vegetative phase change under short day condition. Xu Y. et al. (2016) reported that the precocious phenotype in *brm* was partially suppressed by the mutation of SWN, but no effect by the mutation of CLF. It is unclear why different components of the same PcG complex have distinct target genes and mediate the same developmental process in opposing ways. One possibility is that they form different PRC variants with distinct regulatory roles. Additional experiments are required to resolve this issue. Nonetheless, the aforementioned results confirm that PcG proteins mediate the transition from embryonic to vegetative development, the floral transition, and the juvenile-to-adult phase transition. Our results together with the other reports further indicate that PcG proteins are key players in the control of developmental timing in plants.

## Author Contributions

LM, JL, and ZW designed the experiments; JL, ZW, YH, and YC carried out the experiments; and JL and ZW analyzed the data. LM, JL, and ZW wrote the manuscript. LM supervised the project.

## Conflict of Interest Statement

The authors declare that the research was conducted in the absence of any commercial or financial relationships that could be construed as a potential conflict of interest.
