# Supplementary material for: Polycomb Group Proteins RING1A and RING1B Regulate the Vegetative Phase Transition in Arabidopsis
Source: Front Plant Sci. 2017 May 24;8:867. doi: 10.3389/fpls.2017.00867 (PMC5443144; doi:10.3389/fpls.2017.00867)
Supplement: Supplementary file 1 [file Presentation_1.PDF]

SUPPLEMENTAL DATA

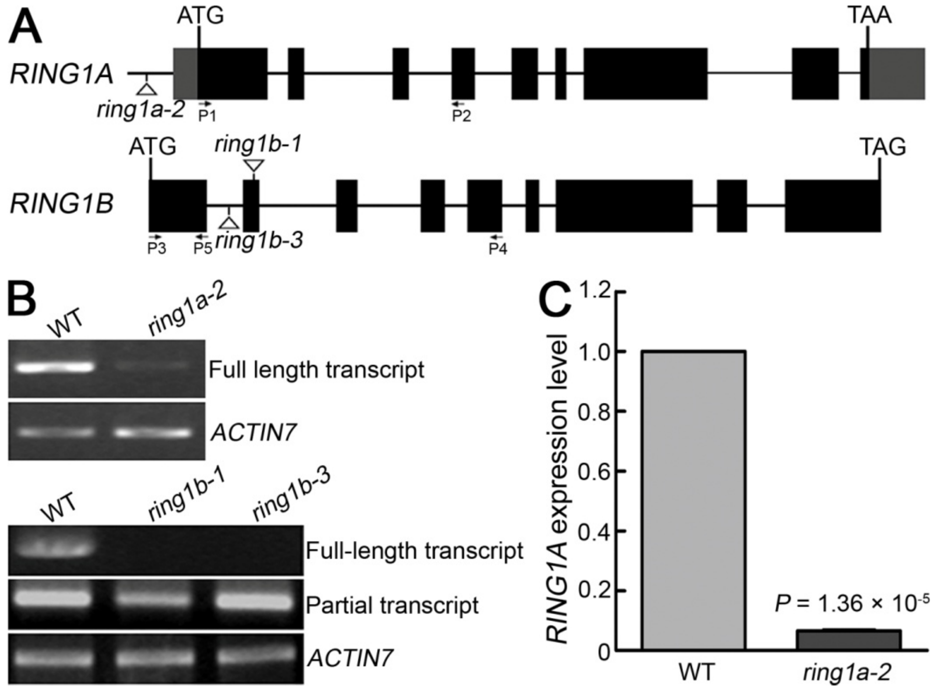

Supplemental Figure 1. Characterization of *ring1a* and *ring1b* mutants. (A) Structures of *RING1A*, *RING1B,* and the T-DNA insertion alleles of the two genes. Exons are represented by filled black boxes, introns by lines, untranslated regions by filled gray boxes, and T-DNA insertions by triangles. The primers used to detect the transcripts of the two genes are indicated as P1–P5. P1/P2 and P3/P4 were used to detect full-length transcripts of *RING1A* and *RING1B,* respectively; P3/P5 was used to detect partial transcripts of *RING1B.* (B) RT-PCR analysis of the *RING1A* or *RING1B* mRNA level in *ring1a-2*, *ring1b-1,* and *ring1b-3* plants using *ACTIN7* as an internal control. (C) Quantitative analysis of the relative expression level of full-length *RING1A* in wild-type and *ring1a-2* plants by real-time RT-PCR. The expression level was normalized to that in wild-type (WT); *ACTIN7* was used as an internal control. The result represents the mean ± SEM of three independent experiments. *p* values of relative mRNA level in *ring1a-2 ring1b-3* versus wild-type were indicated.

Supplemental data Table S1. The primers used for constructions

Name	Sequence (5' to 3')
RING1A Genomic-F	GAGCTC GGTTCGGTGTTCCCTCTGATC
RING1A Genomic-R	GGATCC TGTTAAAAAGTAAAAAAGACTAAGCAGCAG
RING1B Genomic-F	GGATCC TCTTCTCTTCAGGAAATCAACAGTTTG
RING1B Genomic-R	GGTACC CTTGAAAATATACTCTCATGAACTTTGTTC

Supplemental data Table S2. The primers used for RT-PCR analysis

Name	Sequence (5' to 3')
ACTIN-F	AGGCACCTCTTAACCCTAAAGC
ACTIN-R	GGACAACGGAATCTCTCAGC
RING1A-P1	ATGTCTGTCAAGAATAATAGCTTCTCGTC
RING1A-P2	GCAGAACCTGTGGAGACATTCC
RING1B-P3	ATGCCTTCCTTGAAGAGC
RING1B-P4	CCACTACGTCGTGATCTCG
RING1B-P5	CTTCTTCTTCTCCTAACGCTTC

**Supplemental data Table S3. The primers used for real-time PCR in the gene expression assay**	
Name	Sequence (5' to 3')
ACTIN7-F	GGTGTCATGGTTGGTATGGGTC
ACTIN7-R	CCTCTGTGAGTAGAACTGGGTGC
RING1A-F	GAGAAGATGCGGCAATAGAGAATC
RING1A-R	TTGCTTCTTCCGGTAAGCTATG
ARF3-F	TGGTCCCAAGAGAAGCAGG
ARF3-R	TCCACCATCCGAACAAGTG
ARF4-F	GCCGCTGAAGATTGTTTTGCTC
ARF4-R	AGTAGATGCCTCCTTGGTTGACC
pri-miR156a-F	CTCTCCCTCCCTCTCTTTGATTC
pri-miR156a-R	CTCTCCCTCCCTCTCTTTGATTC
SPL3-F	CTTAGCTGGACACAACGAGAGAAGGC
SPL3-R	GAGAAACAGACAGAGACACAGAGGA
SPL9-F	CAAGGTTCAGTTGGTGGAGGA
SPL9-R	TGAAGAAGCTCGCCATGTATTG
SPL10-F	GTGTGGGAGAATGCTCAGGAGG
SPL10-R	ACGGGAGTGTGTTTGATCCCTTGTG

**Supplemental data Table S4. The primers used for real-time PCR in the ChIP assays**	
Name	Sequence (5' to 3')
SPL3P1-F	CTCGAATAACAGAGTTGTGTTTGC
SPL3P1-R	GGTGTAGATGATAAGGTCAATCAAATTA
SPL3P2-F	ACCCGCATGGTGAAATAGC
SPL3P2-R	TGTATCCTTAAAACCTGACGAGGAC
SPL3P3-F	GGCGGAAAAGCACAACTGAC
SPL3P3-R	CAGAGACACAGAGGATTACAAGGAG

SPL9P1-F	GAGAATTATATCCACATGATTTGCTTTC
SPL9P1-R	CTTTAGAACATATTTACACAAGTACGCTTACG
SPL9P2-F	AAGATTAGAAACTGCTACGTCTCTCTC
SPL9P2-R	TTTCGGCTTCCCTGATTTTG
SPL9P3-F	GTAGGAGACAGTACATGGAAGATGAGAAC
SPL9P3-R	ATCAAAAGCCATTATCTGGCAAC
SPL10P1-F	ACCTCCTCATCTTTTCCATTCTC
SPL10P1-R	GACTAATTATGAAACTATTGAAAGAAAATGTC
SPL10P2-F	GGAAGGATGAAATTAATCGAAAGC
SPL10P2-R	GAGAGACCGCCGAAGAAAG
SPL10P3-F	GCTCTCTCTCTTCTGTCAACTACTTCG
SPL10P3-R	AAACTCCATCGGATGATGCAAC
ABI3P1-F	GCCTCCTTACTCACATACAAACCC
ABI3P1-R	TCATCAGCGTCTCCACCGAGTATT
18SP1-F	GCTAACTAGCTACGTGGAGGCATC
18SP1-R	CATCTAAGGGCATCACAGACCTG

Supplemental data Table S5. The probes used for small RNA Northern blotting

Name	Sequence (5' to 3')
miR390	GGCGCTATCCCTCCTGAGCTT
TAS3 *tasi*-RNA	T(+G)GGG(+T)CTT(+A)CAA(+G)GTCA(+A)GAA
miR156	GTGCTCACTCTCTTCTGTCA
U6	AGGGGCCATGCTAATCTTCTC
